# *Lactobacillus sakei* suppresses collagen-induced arthritis and modulates the differentiation of T helper 17 cells and regulatory B cells

**DOI:** 10.1186/s12967-020-02477-8

**Published:** 2020-08-15

**Authors:** Jooyeon Jhun, Hong Ki Min, Jaeyoon Ryu, Seon-Yeong Lee, Jun-Geol Ryu, Jeong Won Choi, Hyun Sik Na, Seung Yoon Lee, Yunju Jung, Sang-Jun Park, Myeong Soo Park, Bin Kwon, Geun Eog Ji, Mi-La Cho, Sung-Hwan Park

**Affiliations:** 1 Rheumatism Research Center, Catholic Research Institute of Medical Science, College of Medicine, The Catholic University of Korea, Seoul, 06591 South Korea; 2 Laboratory of Immune Network, Catholic Research Institute of Medical Science, College of Medicine, College of Medicine, The Catholic University of Korea, Seoul, Republic of Korea; 3 Division of Rheumatology, Department of Internal Medicine, Konkuk University Medical Center, Seoul, Republic of Korea; 4 Research center, BIFIDO Co., Ltd., Hongcheon, Gangwon-do Republic of Korea; 5 Department of Food and Nutrition, Research Institute of Human Ecology, Seoul National University, Seoul, Republic of Korea; 6 Department of Medical Lifescience, College of Medicine, The Catholic University of Korea, 222, Banpo-daero, Seocho-gu, Seoul, 06591 Republic of Korea; 7 Devision of Rheumatology, Department of Internal Medicine, Seoul St. Mary’s Hospital, College of Medicine, The Catholic University of Korea, Seoul, Republic of Korea

**Keywords:** Rheumatoid arthritis, Microbiome, *Lactobacillus sakei*, T helper 17 cell, Regulatory B cell

## Abstract

**Background:**

To evaluate the immunomodulatory effect of *Lactobacillus sakei* in a mouse model of rheumatoid arthritis (RA) and in human immune cells.

**Methods:**

We evaluated whether *L. sakei* reduced the severity of collagen-induced arthritis (CIA) and modulated interleukin (IL)-17 and IL-10 levels, as well as whether it affected the differentiation of CD4^+^ T cells and regulatory B cells. We evaluated osteoclastogenesis after culturing bone marrow-derived mononuclear cells with *L. sakei.*

**Results:**

The differentiation of T helper 17 cells and the serum level of IL-17 were suppressed by *L. sakei* in both human peripheral blood mononuclear cells and mouse splenocytes. The serum level of IL-10 was significantly increased in the *L. sakei*-treated group, whereas the regulatory T cell population was unchanged. The population of regulatory B cells significantly increased the in *L. sakei*-treated group. Oral administration of *L. sakei* reduced the arthritis incidence and score in mice with CIA. Finally, osteoclastogenesis and the mRNA levels of osteoclast-related genes were suppressed in the *L. sakei*-treated group.

**Conclusion:**

*L. sakei* exerted an anti-inflammatory effect in an animal model of RA, regulated Th17 and regulatory B cell differentiation, and suppressed osteoclastogenesis. Our findings suggest that *L. sakei* has therapeutic potential for RA.

## Background

Rheumatoid arthritis (RA) is a chronic destructive autoimmune-mediated arthritis. The prevalence of RA ranges from 0.1% to 5%, depending on ethnicity and sex [1, 2]. Treatments target the attenuation of arthralgia, as well as the suppression of joint destruction, which is irreversible and reduces the quality of life in affected patients. Multiple factors contribute to the development and progression of RA [3]. The interaction between the host and the microbiota is a key modulator of the immune response and may contribute to autoimmune diseases, including RA [4]. The first evidence of a role for the microbiome in the pathogenesis of RA involved the observation of a higher frequency of *Porphyromonas gingivalis* (the etiologic agent of periodontitis) in patients with RA, compared with healthy controls [5]. Microbes reside at various body sites, particularly those exposed to the external environment (e.g., oral cavity, respiratory tract, and gastrointestinal tract). Approximately 100 trillion microbes reside in the human body, primarily in the gastrointestinal tract [4]. Dysbiosis in patients with RA, as well as following an inflammatory response in the gastrointestinal tract, is a key immunologic mechanism that contributes to the pathogenesis of RA [6, 7]. Therefore, controlling the gastrointestinal tract microbiome via oral administration of probiotics has therapeutic potential for RA. However, oral probiotics reportedly have minimal clinical efficacy in patients with RA [8]; notably, the prior study was limited by its small sample size and the use of various strains and quantities of probiotics. Few studies have demonstrated the therapeutic potential of probiotics in animal models of RA [9–11].

The genus *Lactobacillus* is an important component of the microbiomes of the human gastrointestinal tract and urinary tract*. Lactobacillus sakei* is typically found in cooked ham and inhibits pathogenic microbes such as *Escherichia coli* O157:H7 and *Listeria monocytogenes* [12]. In a mouse model, oral administration of *L. sakei* attenuated colitis and reduced the expression of proinflammatory cytokines [13]. Furthermore, *L. sakei* has been shown to ameliorate obesity and reduce the levels of biomarkers of obesity (e.g., leptin) in mice [14], and it can suppress pathologic bacterium, *Corynebacterium tuberculostearicum,* which induces chronic rhinosinusitis in human [15]. *L. sakei* is widely found in fermented food such as kimchi, yogurt, and cheese [14, 16] and commonly used for meat preservation [17], which makes feasible to translate result of animal study into human application (translation of T1 research to T2 research). Results from the preclinical animal study can suggest the basic mechanism of *L. sakei* how they exert anti-inflammatory effects and encourage future clinical trials for applying *L. sakei* to real patients. Furthermore, common food sources of *L. sakei* increase the possibility to apply *L. sakei* in human subjects. The composition and diversity of gut microbiome differs depending on the immune status, and concomitant anti-rheumatic medication such as methotrexate could alter composition of microbiome and its influence on functional potency [18, 19]. *L. sakei* has clear anti-inflammatory activity, but its effect on RA is unclear. In addition, the anti-inflammatory potency of *L. sakei* from healthy and RA patients may differ.

A variety of immune cells and proinflammatory cytokines play important roles in the pathogenesis of RA [20]. T helper 17 cells (Th17) are implicated in several inflammatory and immune-mediated diseases, including RA; these cells produce interleukin (IL)-17 [21], which promotes synovitis, contributes to joint destruction, and augments osteoclastogenesis. Therefore, inhibition of IL-17 is a goal in the treatment of chronic inflammatory diseases, including RA [21, 22]. IL-10 is a key anti-inflammatory/immunomodulatory cytokine [23] produced by various immune cells including regulatory T cells (Tregs), macrophages, dendritic cells, and B cells. B cells are the progenitors of plasma cells, and B/plasma cells mediate RA by producing autoantibodies. However, B cells can also modulate immunity by producing anti- and pro-inflammatory cytokines [24]. Regulatory B cells (Bregs) secrete IL-10, transforming growth factor-β, and IL-35; moreover, they suppress the differentiation of proinflammatory lymphocytes, including Th17 and cytotoxic CD8^+^ T cells [25]. The numbers and activities of IL-10–producing Bregs are reduced in patients with RA [26]. Several animal models for RA have been established, and collagen-induced arthritis (CIA) model resembles RA symptom by inducing autoantibody and Th17 polarization [27]. CIA model was suitable to evaluate immune response of *L. sakei* on T cell and B cell response.

In this in vitro study, we evaluated the immunologic responses to *L. sakei* by human and animal cells. Specifically, we assessed the serum levels of cytokines, as well as the differentiation of T and B lymphocytes and osteoclasts. Furthermore, we analyzed the effects of *L. sakei* on collagen-induced arthritis (CIA) by administrating *L. sakei* extracted from healthy control and RA patient.

## Methods

### Animals

Seven-week-old male DBA/1 J mice (Orient Bio, Gyeonggi-do, Republic of Korea) were maintained under specific pathogen-free conditions and provided standard laboratory mouse chow (Ralston Purina, St. Louis, MO, USA) and water ad libitum. The mice were housed at five per cage in a room maintained under controlled temperature (21–22 °C) and lighting (12/12 h light/dark cycle) conditions. All experimental procedures were approved by the Institutional Animal Care and Use Committee of the School of Medicine and the Animal Research Ethics Committee of the Catholic University of Korea; the procedures complied with the Laboratory Animals Welfare Act, in accordance with the Guide for the Care and Use of Laboratory Animals.

### Induction and treatment of arthritis

To induce CIA in DBA/1 J mice, chicken type II collagen (4 mg/mL) was dissolved overnight in 0.1 N acetic acid with gentle rotation at 4 °C. DBA/1 J mice were injected intradermally at the base of the tail with 100 µg of chicken type II collagen; an emulsion of Freund’s adjuvant (Difco, Detroit, MI, USA) was administered to the hind legs of the mice as a booster injection. To assess the effects of *L. sakei* on the severity of CIA, DBA/1 J mice received 50 mg/kg *L. sakei* in saline, or vehicle alone, via oral gavage six times per week for 7 weeks; this treatment began on day 21 after the primary immunization.

### Clinical assessment of arthritis

The severity of arthritis was evaluated by three independent observers. The mice were observed twice weekly to determine the onset and severity of joint inflammation for up to 7 weeks after the primary immunization. The severity of arthritis was assessed on a scale of 0–4, based on the following criteria [28]: 0 = no edema or swelling; 1 = slight edema, with erythema limited to the foot or ankle; 2 = slight edema, with erythema from the ankle to the tarsal bone; 3 = moderate edema, with erythema from the ankle to the tarsal bone; and 4 = severe edema, with erythema from the ankle to the entire leg. The arthritis score of each mouse was calculated as the sum of the scores of the four limbs; the highest possible arthritis score for each mouse was 16. The mean arthritis index was used to compare the scores of the control and experimental groups.

### Histological analysis

Joint tissues were fixed in 10% (v/v) neutral-buffered formalin, decalcified in a histological decalcifying agent (Calci-Clear Rapid; National Diagnostics, Atlanta, GA, USA), embedded in paraffin, and cut into 5-µm-thick sections. The sections were stained with hematoxylin and eosin, as well as Safranin O, to detect proteoglycans. Inflammation was scored using the following criteria: 0 = no inflammation; 1 = slight thickening of the lining, or infiltration of some cells into the underlying layer; 2 = slight thickening of the lining, with infiltration of some cells into the underlying layer; 3 = thickening of the lining, with influx of cells into the underlying layer and cells evident in the synovial space; and 4 = extensive infiltration of the synovium by inflammatory cells. Cartilage damage was evaluated by staining with Safranin O and toluidine blue, and the extent of damage was scored as follows: 0 = no destruction; 1 = minimal erosion (limited to single spots); 2 = slight-to-moderate erosion in a limited area; 3 = more extensive erosion; and 4 = general destruction.

### Immunohistopathological analysis of arthritis

Joint tissue was first incubated with primary antibodies against tumor necrosis factor-α, IL-1β (R&D Systems, Minneapolis, MN, USA),, IL-6(R&D Systems),, and IL-17 (R&D Systems) overnight at 4 °C. Samples were incubated with a biotinylated secondary antibody, followed by incubation with a streptavidin–peroxidase complex for 1 h. Samples were then developed using chromogen 3,3′-diaminobenzidine (Thermo Scientific, Rockford, IL,USA). The sections were examined under a photomicroscope (Olympus, Tokyo, Japan). The number of positive cells was counted using Adobe Photoshop software (Adobe, USA) on high-power digital image (magnification: 400). Positive cells were enumerated visually by three individuals, and the mean values were calculated.

### Enzyme-linked immunosorbent assay

The IL-17 and IL-10 concentrations in the supernatants of cultures of human cells were measured by sandwich enzyme-linked immunosorbent assay (ELISA) (R&D Systems). The absorbance at 405 nm was determined using an ELISA microplate reader (Molecular Devices, Sunnyvale, CA, USA).

### Isolation and stimulation of splenocytes

Splenocytes were prepared from the spleens of normal C57BL6 mice. Splenocytes were maintained in Roswell Park Memorial Institute (RPMI)-1640 medium supplemented with 5% fetal bovine serum (Gibco, Grand Island, NY, USA) before stimulation with plate-bound anti-CD3 (0.5 µg/mL) for 3 days; they were then subjected to flow cytometry analysis.

### Isolation and stimulation of peripheral blood mononuclear cells

Peripheral blood mononuclear cells (PBMCs) were prepared from heparinized blood by standard Ficoll–Paque density gradient centrifugation (GE Healthcare Biosciences, Uppsala, Sweden). Cells were cultured in RPMI-1640 medium (Gibco BRL, Carlsbad, CA, USA) containing penicillin (100 U/mL), streptomycin (100 μg/mL), and 10% fetal bovine serum (Gibco BRL) that had been inactivated by heating to 55 °C for 30 min. Suspensions of both cell types were dispensed into 48-well plates (Nunc, Rosklide, Denmark). PBMCs were incubated with plate bound anti-CD3 (0.5 µg/mL) or lipopolysaccharide 100 ng/mL for 3 days.

### Preparation of bacteria

Patients with RA fulfilled the 2010 American College of Rheumatology and European League Against Rheumatism classification criteria [29]. Bacterial genomic DNA was isolated from fecal samples of RA patients (RH1114) and healthy normal subjects (RH1117). The study design was approved by the Institutional Review Board of Seoul St. Mary’s Hospital, The Catholic University of Korea (approval ID: KC17TNSI0570). Written informed consents were obtained from all study participants.

50 µL of each bacterium was inoculated to 5 L cMRS liquid medium and incubated for 20 h at 37 °C. After incubation, in order to remove the medium component, the supernatant of the medium was discarded by centrifugation (High Speed Centrifuge, 2236HR, Korea) at 20 °C and 7000 rpm with phosphate buffered saline (PBS) washing, twice. The washed bacterial cells were powdered by drying the remaining medium components at 36 °C, 2000 rpm for 24 h using Scanvac Speed Vacuum Concentrator (Labogene Aps, Lillerød, Denmark). The powdered bacterial cells were heat-inactivated by heating at 80 °C for 30 min. All bacteria used in this study were isolated from healthy subjects and patients with RA.

### Flow cytometry

Levels of cytokines and transcription factors were assessed by intracellular staining using anti-IL-17-FITC, anti-Foxp3-FITC, and anti-Foxp3-PE antibodies (all from eBioscience, San Diego, CA, USA). Cells were stimulated with phorbol myristate acetate and ionomycin with the addition of GolgiStop for 4 h. Cultured cells were surface labeled for 30 min and permeabilized with Cytofix/Cytoperm solution (BD Pharmingen, Heidelberg, Germany). Cells were intracellularly stained with fluorescent antibodies and subjected to flow cytometry (FACSCalibur; BD Biosciences, Franklin Lakes, NJ, USA). Events were collected and analyzed using FlowJo software (Tree Star, Ashland, OR, USA).

### In vitro osteoclastogenesis and tartrate-resistant acid phosphatase staining

Bone marrow cells from mouse femurs were cultured in alpha-minimal essential medium (Invitrogen, Carlsbad, CA, USA) containing antibiotics and 10% heat-inactivated fetal bovine serum to separate floating and adherent cells. Nonadherent cells were removed by washing with media, and preosteoclasts were cultured in the presence of 10 ng/mL macrophage colony-stimulating factor, 100 ng/mL receptor activator of nuclear factor kappa-Β (RANK) ligand (RANKL) (PeptoTech, London, UK), and *L. sakei* for 4 days to generate osteoclasts. The medium was changed every 2 days. Osteoclasts were generated after 8–10 days.

### TRAP staining

A commercial TRAP kit (Sigma-Aldrich) was used according to the manufacturer’s instructions; however, counterstaining with hematoxylin was not performed. TRAP-positive multinuclear cells (MNCs) containing three or more nuclei were counted as osteoclasts.

### Real-time polymerase chain reaction

Polymerase chain reaction was performed using a Light Cycler 2.0 instrument (Roche Diagnostics, Mannheim, Germany) with software version 4.0. All reactions were performed using Light Cycler Fast Start DNA Master SYBR Green I (TaKaRa, Shiga, Japan), in accordance with the manufacturer’s instructions. The following primers were used: TRAP, 5′-TCC TGG CTC AAA AAG CAG TT-3′ (sense) and 5′-ACA TAG CCC ACA CCG TTC TC-3′ (antisense); calcitonin receptor, 5′-CGG ACT TTG ACA CAG AA-3′ (sense) and 5′-AGC AAT CGA CAA GGA GT-3′ (antisense); integrin b3, 5′-CTG TGG GCT TTA AGG ACA GC-3′ (sense) and 5′-GAG GGT CGG TAA TCC TC-3′ (antisense); cathepsin K, 5′-CAG AGG TGT GTA CTA TG-3′ (sense) and 5′-GCG TTG TTC TTA TTC CGA GC-3′ (antisense); RIPK1, 5′-CTG TTC CCT GTG CCC AAT AA-3′ (sense) and 5′-ATG ACT CTG AAG CTG TCC TTT C-3′ (antisense); and RIPK3, 5′-GCA CTC CTC AGA TTC CAC ATA C-3′ (sense) and 5′-GTG TCT TCC ATC TCC CTG ATT C-3′ (antisense).

### Statistical analysis

Statistical analysis was performed using the nonparametric Mann–Whitney U test for comparisons of two groups, and one-way analysis of variance with the Bonferroni post hoc test for multiple groups. Prism ver. 5.01 software (GraphPad Software Inc., San Diego, CA, USA) was used. *P* < 0.05 was regarded as the threshold for statistical significance. Data are presented as means ± standard deviations.

## Results

### Effect of *L. sakei* on helper T cell differentiation and cytokine production

**Fig. 1:**
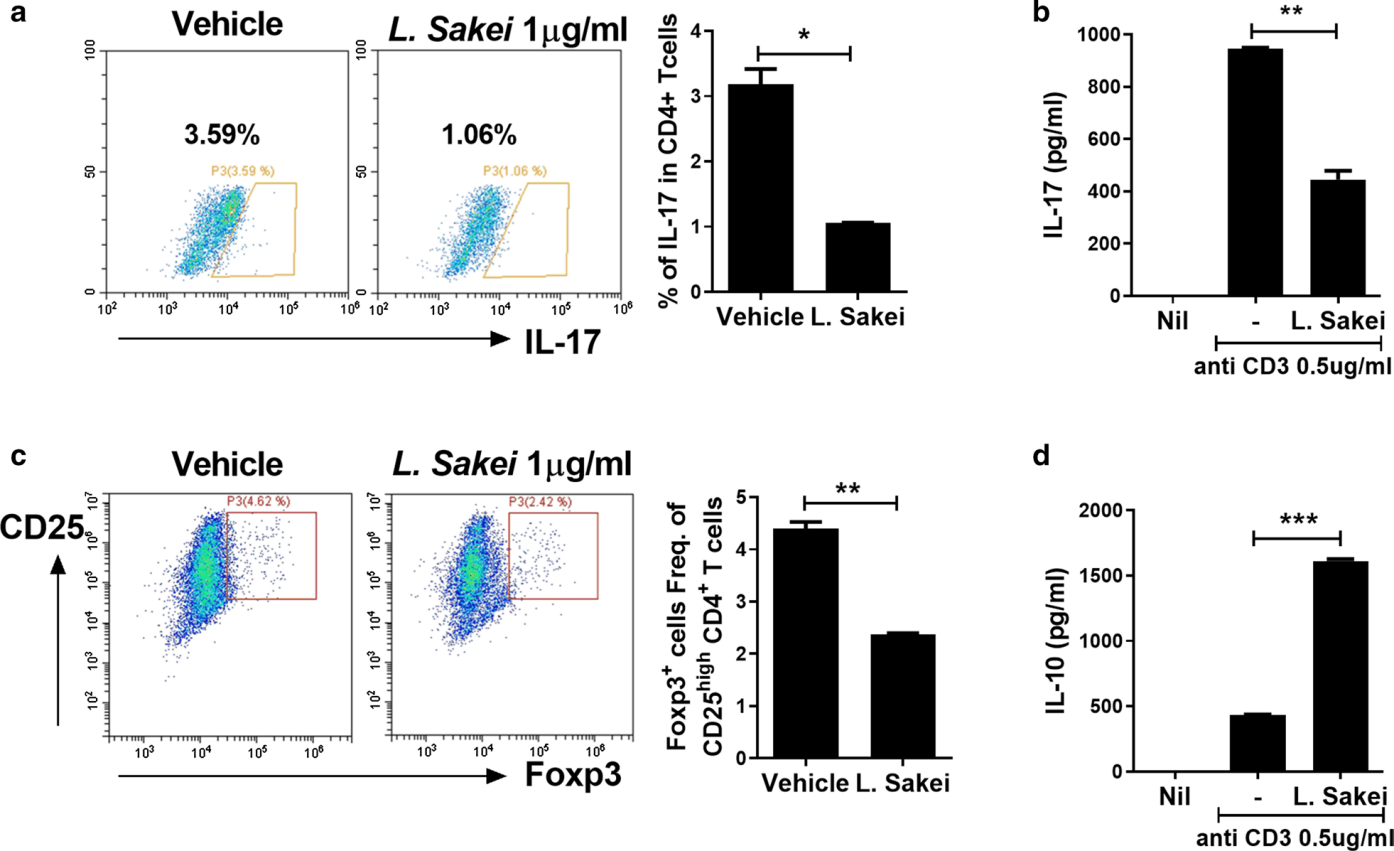
*L. sakei* suppresses Th17 differentiation and IL-17 expression in mouse splenocytes. **a** C57bl/6 splenocytes were cultured with an anti-CD3 antibody for 72 h and the resulting CD4^+^IL-17^+^ and CD4^+^CD25^high^Foxp3^+^ cells were enumerated. **b** IL-17 and IL-10 levels in culture supernatant measured by ELISA. Data are means ± standard deviations from three independent experiments (*P < 0.05, **P < 0.03, ***P < 0.01)

To evaluate the effects of *L. sakei* on CD4^+^ T cell differentiation and cytokine production, splenocytes from C57BL/6 mice were cultured with vehicle (control) or *L. sakei* (1 μg/mL). The Th17 population (IL-17^+^CD4^+^ T cells) and the IL-17 level in culture supernatant were significantly reduced by exposure to *L. sakei* (Fig. 1a, b). In the *L. sakei*-treated group, the Treg (Foxp3^+^CD25^high^CD4^+^ T cells) population was suppressed, whereas the IL-10 level was increased (Fig. 1c, d). The Th17 population and IL-17 level were suppressed by exposure to *L. sakei*; however, the IL-10 level was increased.

**Fig. 2:**
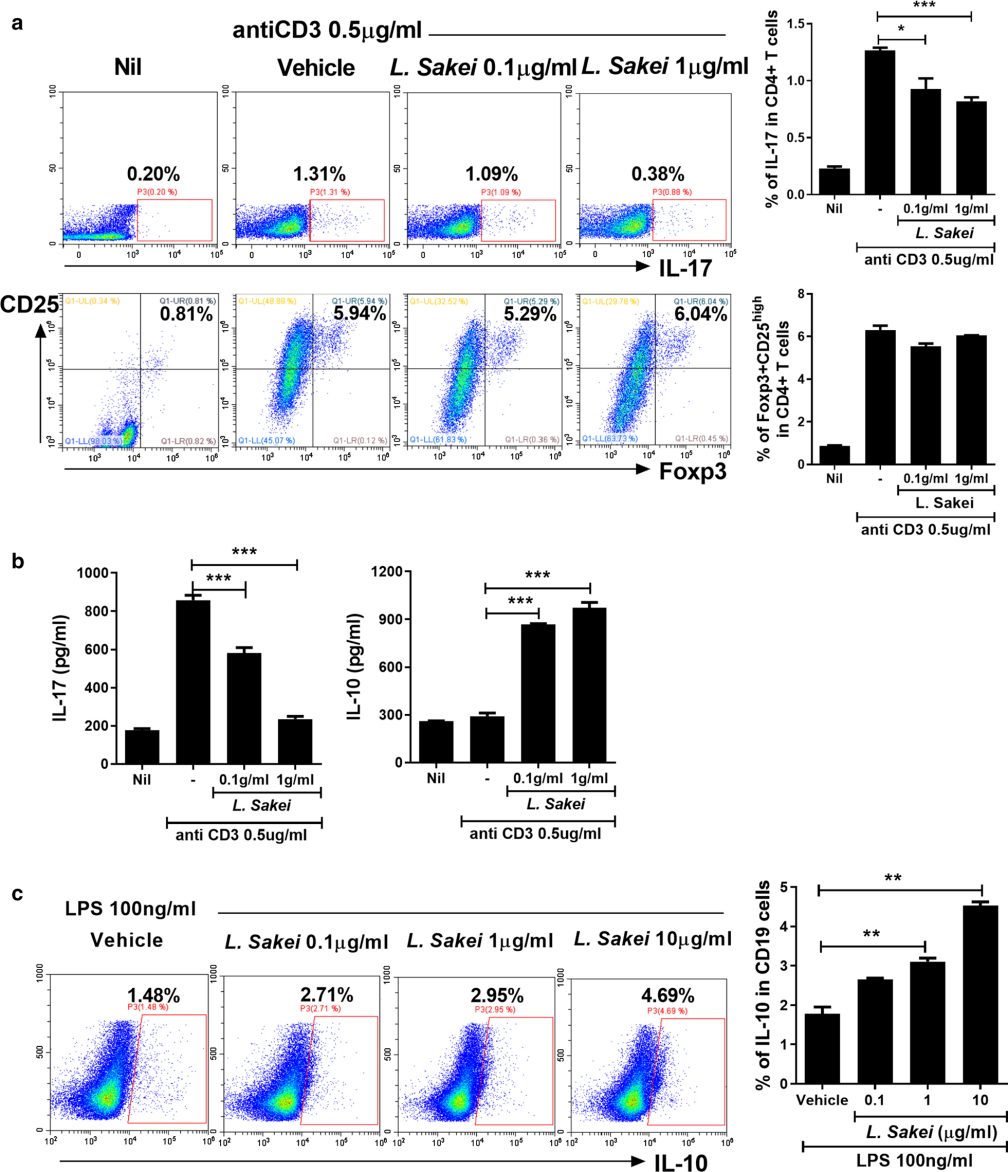
*L. sakei* increases the IL-10 level and reduces the Th17 population and IL-17 expression in human PBMCs. **a** Normal human PBMCs were cultured with an anti-CD3 antibody for 72 h and the resulting CD4^+^IL-17^+^ and CD4^+^CD25^high^Foxp3^+^ cells were enumerated. **b** IL-17 and IL-10 levels in culture supernatant measured by ELISA. **c** Normal human PBMCs were cultured in the presence of lipopolysaccharide for 72 h and the resulting CD19^+^IL-10^+^ cells were enumerated. Data are means ± standard deviations from three independent experiments (*P < 0.05, **P < 0.03, ***P < 0.01)

To evaluate the effects of *L. sakei* on the differentiation of human CD4^+^ T cells and identify the mechanism for increased levels of IL-10, we cultured human PBMCs in the presence of vehicle or *L. sakei* (0.1 or 1 μg/mL). Exposure to *L. sakei* reduced the Th17 population in a dose-dependent manner, whereas the Treg population was unaffected (Fig. 2a). Exposure to *L. sakei* also reduced the IL-17 level and increased the level of IL-10 (Fig. 2b). Furthermore, treatments with 1 or 10 μg/mL *L. sakei* resulted in an increased Breg population, compared to the control (Fig. 2c). Therefore, we concluded that *L. sakei* exerts an immunomodulatory effect by altering the populations of Th17 and Breg cells, as well as the levels of IL-17 and IL-10.

### *sakei*-mediated inhibition of the development of CIA

**Fig. 3:**
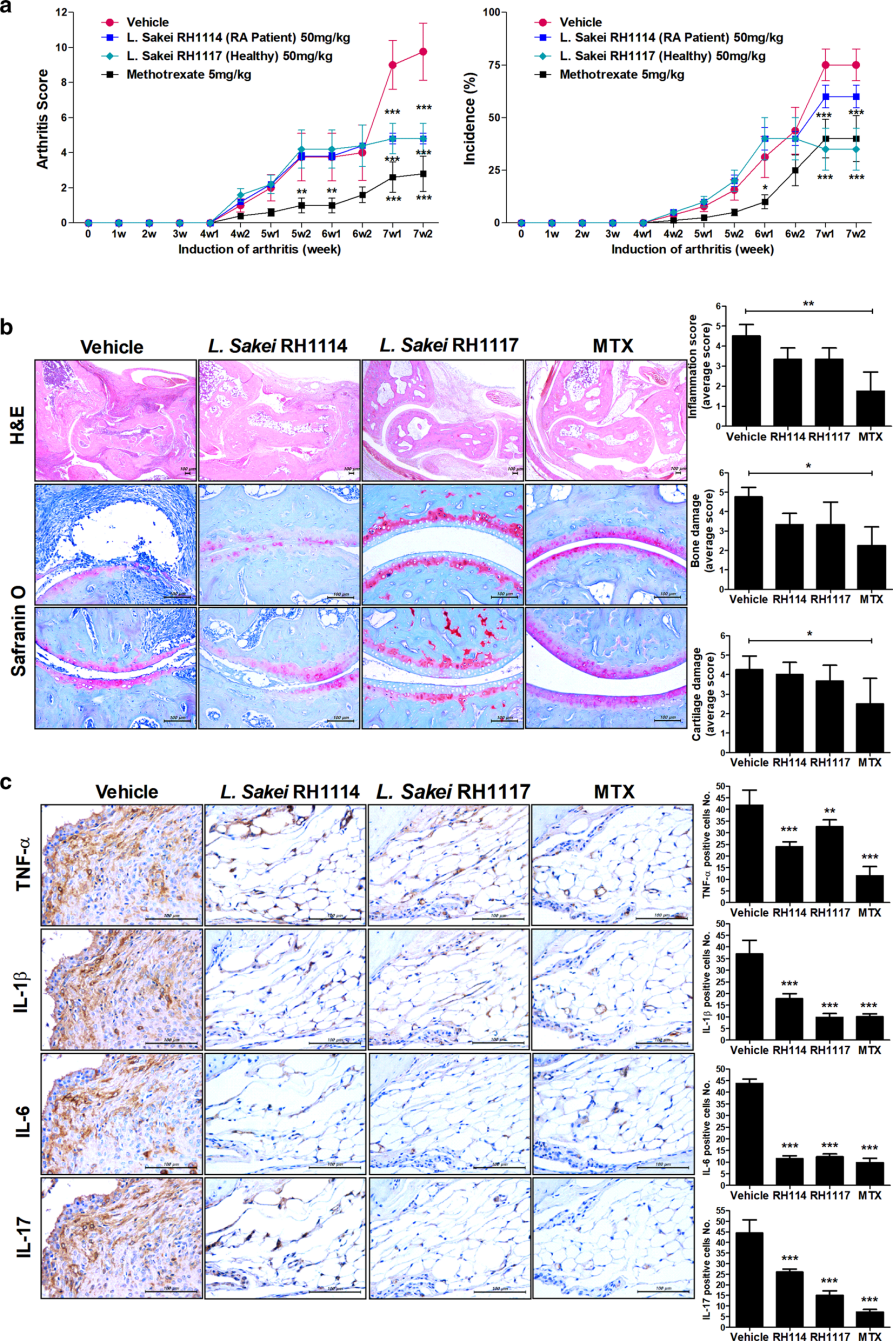
*L. sakei* RH1114 and RH1117 inhibit the development of RA in a mouse model. **a** *L. sakei* (50 mg/kg) (n = 5) was fed orally once per day to DBA/1 J mice with CIA. Left, arthritis score; right, arthritis incidence. **b** Effect of *L. sakei* on RA in mice with CIA. Tissue from the hind-paw joints was stained with hematoxylin and eosin, as well as Safranin O. **c** Immunohistochemical analysis showed that *L. sakei* ameliorated CIA and downregulated proinflammatory factors, compared to vehicle (control). Immunohistochemical staining for tumor necrosis factor-α, IL-1β, IL-6, and IL-17 in the synovium of mice with CIA (scale bar, 100 µm) (*P < 0.05, **P < 0.03, ***P < 0.01)

**Fig. 4:**
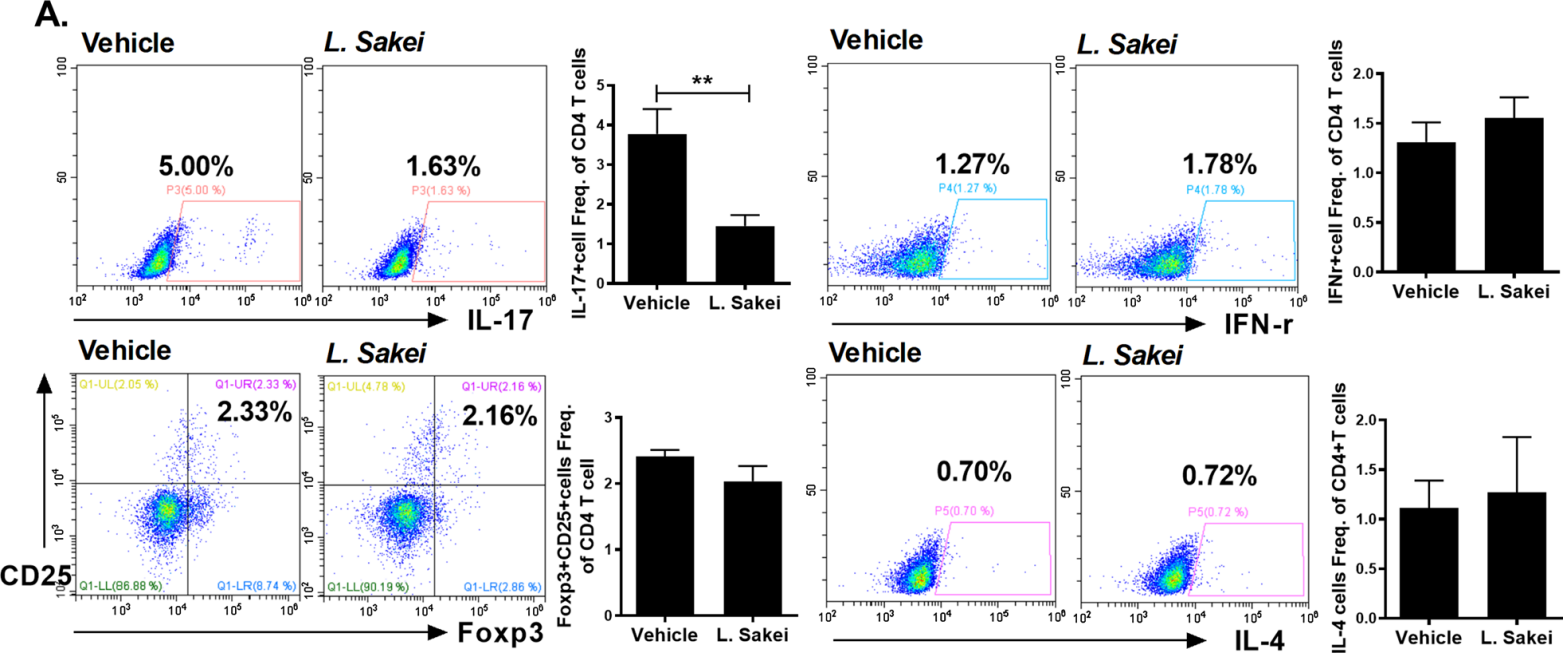
*L. sakei* reduces IL-17 expression but maintains expression of Foxp3 in CD4^+^ T cells from the spleen of mice with CIA. **a.** Flow cytometry of Th1 cells (IFN-r^+^CD4^+^), Th2 cells (IL-4^+^CD4^+^), Th17 cells (CD4^+^IL17^+^), and Tregs (Foxp3^+^CD25^+^CD4^+^) from the spleen of mice with CIA

Oral administration of *L. sakei* RH1114 or RH1117 to CIA mice attenuated CIA, as evidenced by a significantly lower arthritis score compared to the control mice. Only *L. sakei* RH1117 significantly reduced the incidence of CIA (Fig. 3a). In mice that received *L. sakei*, the severity of arthritis was attenuated and cartilage preservation was enhanced, compared to the control mice (Fig. 3b). Furthermore, immunohistochemical staining of hind joints showed that administration of *L. sakei* reduced the levels of tumor necrosis factor-α, IL-1β, IL-6, and IL-17 (Fig. 3c). Flow cytometry analysis of splenocytes showed that the Th17 (IL17^+^CD4^+^ T cells) population was reduced by administration of *L. sakei*, whereas the populations of Th1 (IFN-r^+^CD4^+^ T cells), Th2 (IL-4^+^CD4^+^ T cells), and Tregs (Foxp3^+^CD25^+^CD4^+^ T cells) did not differ significantly between *L. sakei*- and vehicle-treated mice with CIA (Fig. 4).

### Effects of *L. sakei* on osteoclastogenesis

**Fig. 5:**
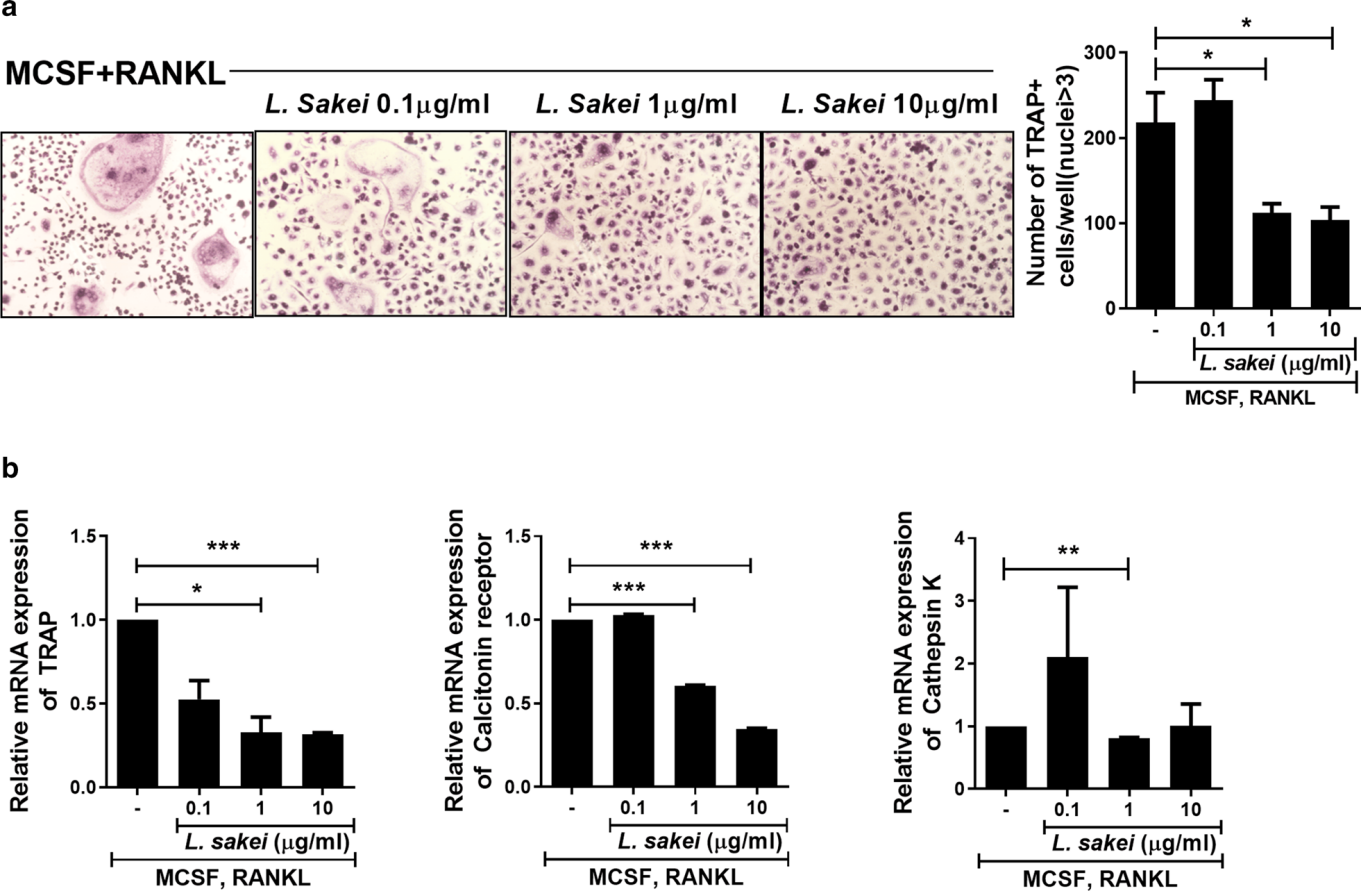
*L. sakei* inhibits osteoclastogenesis. **a** Suppression of TRAP in the ankle joints of *L. sakei*-treated mice with autoimmune arthritis at 7 weeks, demonstrated by immunohistochemical staining. **b** TRAP staining of human monocytes cultured with 25 ng/mL macrophage colony-stimulating factor and 30 ng/mL RANKL in the presence or absence of *L. sakei* for 9 days. **c** mRNA levels of TRAP, calcitonin receptor, and cathepsin K assessed by real-time polymerase chain reaction and normalized to the level of β-actin. Bars indicate means ± standard errors of the mean (*P < 0.05, **P < 0.03, ***P < 0.01)

TRAP-positive multinucleated cells containing three or more nuclei were regarded as osteoclasts. *L. sakei* reduced the numbers of TRAP-, RANK-, and RANKL-positive cells in mice with CIA. The numbers of TRAP^+^ multinucleated cells were significantly lower in mice that received 1 or 10 μg/mL *L. sakei* (Fig. 5a). Similarly, the expression levels of osteoclast-related genes (TRAP and calcitonin receptor) were reduced by administration of 1 or 10 μg/mL *L. sakei* (Fig. 5b). The expression of cathepsin K was significantly reduced only by administration of 1 μg/mL *L. sakei*.

## Discussion

This is the first report of the anti-inflammatory and immunomodulatory activity of *L. sakei* in a mouse model of RA. Administration of *L. sakei* altered the IL-17/IL-10 balance by regulating the Th17 and Breg populations, but not the Treg population. In addition, oral administration of *L. sakei* reduced the incidence and severity of arthritis and suppressed osteoclastogenesis.

Disruption of normal-flora homeostasis (i.e., dysbiosis) has important immunologic sequalae. In health, the host–microbiome interaction maintains homeostasis. However, dysbiosis has been implicated in several autoimmune diseases [4]. In patients with RA, periodontitis caused by *P. gingivalis* is reportedly associated with the production of anti-citrullinated protein antibodies [30]; moreover, 16S rRNA sequencing of gut microbiota showed an increased population of *Prevotella copri* in these patients [18, 31]. Furthermore, *P. copri* isolated from patients with RA induced the differentiation of Th17 cells [31]. These results indicate a role for the microbiome in the onset of RA. In this study, *L. sakei* modulated the Th17 and Breg populations and attenuated arthritis in mice with CIA. Therefore, administration of *L. sakei* has therapeutic potential for RA.

Proinflammatory cytokines and their source cells are implicated in the pathogenesis of RA; thus, modulation of these cytokines and cells is a target of treatment for RA [20]. Suppression of the inflammatory response with concurrent enhancement of the anti-inflammatory response is the ideal therapeutic approach for patients with autoimmune diseases. Patients with RA have an increased population of Th17 cells and an elevated serum level of IL-17 [32, 33]; modulation of these components has been reported to exert beneficial effects in several animal models of RA [34, 35]. In the context of RA, B cells and plasma cells were previously considered to be limited to the production of autoantibodies, such as anti-rheumatoid factor and anti-citrullinated protein antibodies. However, Bregs also produce anti-inflammatory cytokines and suppress the expansion of pathogenic T cells [25]. In patients with RA, the numbers and activities of IL-10–producing Bregs are reduced, compared to healthy controls [36]. In addition, targeting proinflammatory cytokines, such as TNF-α and IL-6, have shown clinical improvement in RA patients [20], and our results showed decrement of these cytokines in affected joints. In the present study, administration of *L. sakei* reduced pathologic cytokines in peripheral joints of CIA animal, and the population of Th17 cells and serum level of IL-17, whereas increased the population of Breg cells and serum level of IL-10.

Difference in composition of gut microbiome in RA is well known, and even disease-modifying antirheumatic drug can change the functional potency of microbiome. We hypothesized that same bacterium species may have different potential on anti-inflammatory effect according to disease status. In present study, the *L. sakei* RH1114 (from RA patients) did not show overall decrease in arthritis incidence in CIA model, whereas *L. sakei* RH1117 (from healthy control) showed obvious decline in arthritis incidence. Present study showed the functional difference between *L. sakei* from healthy donor and RA patients in animal model, and further study implicating these differences in human study may clarify the functional difference of *L. sakei* depending on the source.

Probiotics have fewer side effects than conventional or biologic disease-modifying antirheumatic drugs, because they aim to restore the normal microbial environment and immune system. *L. sakei* has been reported to exert beneficial effects on several inflammatory disorders such as colitis, atopic dermatitis, chronic rhinosinusitis, and obesity [13–15, 37]. Although the preventive effect of *L. sakei* on CIA was smaller than that of methotrexate, it was greater than the effect of vehicle. Increased osteoclastogenesis promotes joint destruction and early onset osteoporosis in patients with RA [20]. Treatment of early onset osteoporosis is important in these patients, because their chronic inflammatory status (due to long-term use of glucocorticoids) can exacerbate osteoporosis [38]. Osteoclasts are the main effector cells in osteoporosis, and a therapeutic modality that acts on both arthritis and osteoporosis would likely be effective for patients RA. In this context, oral administration of *L. sakei* in combination with conventional/biologic disease-modifying antirheumatic drug therapy is promising. However, translational study on human (established RA and high-risk population for RA) could clarify the therapeutic and preventive effect of L. sakei in real RA patients.

## Conclusion

This study showed that administration of *L. sakei* exerted a beneficial effect in an animal model of RA. Furthermore, *L. sakei* skewed the Th17 and Breg populations towards an anti-inflammatory phenotype and suppressed osteoclastogenesis. Therefore, *L. sakei* has therapeutic potential for RA.

## Data Availability

All data are available in the manuscript or upon request to the authors.

## Abbreviations

CIA: Collagen induced arthritis
RA: Rheumatoid arthritis
IL: Interleukine
Treg: T regulatory
TRAP: Tartrate-resistant acid phosphatase
RANK: Receptor activator of nuclear factor kappa-B
RANKL: RANK ligand
M-CSF: Macrophage colony-stimulating factor
